# Supplementary material for: Integrative Multi-Omics and Network Analyses Reveal Pathogenic and Protective Pathways in Centronuclear Myopathies
Source: Int J Mol Sci. 2025 Nov 28;26(23):11572. doi: 10.3390/ijms262311572 (PMC12691857; doi:10.3390/ijms262311572)
Supplement: Supplementary file 1 [file ijms-26-11572-s001.zip › 251120_U02_Supplementary/250819_SuppInfo_S1.pdf]

HPO term to parent SPARQL query:

```
PREFIX xsd: <http://www.w3.org/2001/XMLSchema#>
PREFIX rdf: <http://www.w3.org/1999/02/22-rdf-syntax-ns#>
PREFIX rdfs: <http://www.w3.org/2000/01/rdf-schema#>
PREFIX GO: <http://www.geneontology.org/formats/oboInOwl#>
SELECT DISTINCT ?term_ID ?term_label ?parent_ID ?parent_label  WHERE {
  ?root GO:id "HP:0000001"^^xsd:string .
  ?term rdfs:subClassOf ?parent ;
       rdfs:subClassOf* ?root ;
       rdfs:label ?term_label ;
       GO:id ?term_ID .
  ?parent rdfs:label ?parent_label ;
        GO:id ?parent_ID
}
```

HPO term to tissue SPARQL query:

```
PREFIX xsd: <http://www.w3.org/2001/XMLSchema#>
PREFIX owl: <http://www.w3.org/2002/07/owl#>
PREFIX rdf: <http://www.w3.org/1999/02/22-rdf-syntax-ns#>
PREFIX rdfs: <http://www.w3.org/2000/01/rdf-schema#>
PREFIX GO: <http://www.geneontology.org/formats/oboInOwl#>
PREFIX obo: <http://purl.obolibrary.org/obo/>

SELECT DISTINCT ?hpo_id ?label ?class_id ?class_label WHERE {
  VALUES ?class_id {"HP:0009124"^^xsd:string "HP:0000834"^^xsd:string "HP:0012443"^^xsd:string
"HP:0004297"^^xsd:string "HP:0000014"^^xsd:string "HP:0001871"^^xsd:string
"HP:0005561"^^xsd:string "HP:0002763"^^xsd:string "HP:0000951"^^xsd:string
"HP:0000478"^^xsd:string "HP:0005264"^^xsd:string "HP:0001627"^^xsd:string
"HP:0002242"^^xsd:string "HP:0000077"^^xsd:string "HP:0001392"^^xsd:string
"HP:0002088"^^xsd:string "HP:0000137"^^xsd:string "HP:0001732"^^xsd:string
"HP:0000828"^^xsd:string "HP:0001194"^^xsd:string "HP:0008775"^^xsd:string
"HP:0011805"^^xsd:string "HP:0030935"^^xsd:string "HP:0001743"^^xsd:string
"HP:0000035"^^xsd:string "HP:0000820"^^xsd:string "HP:0003110"^^xsd:string
"HP:0002597"^^xsd:string "HP:0002031"^^xsd:string "HP:0000769"^^xsd:string
"HP:0002577"^^xsd:string}
  ?term GO:id ?hpo_id ;
       rdfs:label ?label ;
       rdfs:subClassOf+ ?class .
  ?class GO:id ?class_id;
        rdfs:label ?class_label .
}
```
