# Supplementary figures and images for: Integrative Multi-Omics and Network Analyses Reveal Pathogenic and Protective Pathways in Centronuclear Myopathies
Source: Int J Mol Sci. 2025 Nov 28;26(23):11572. doi: 10.3390/ijms262311572 (PMC12691857; doi:10.3390/ijms262311572)

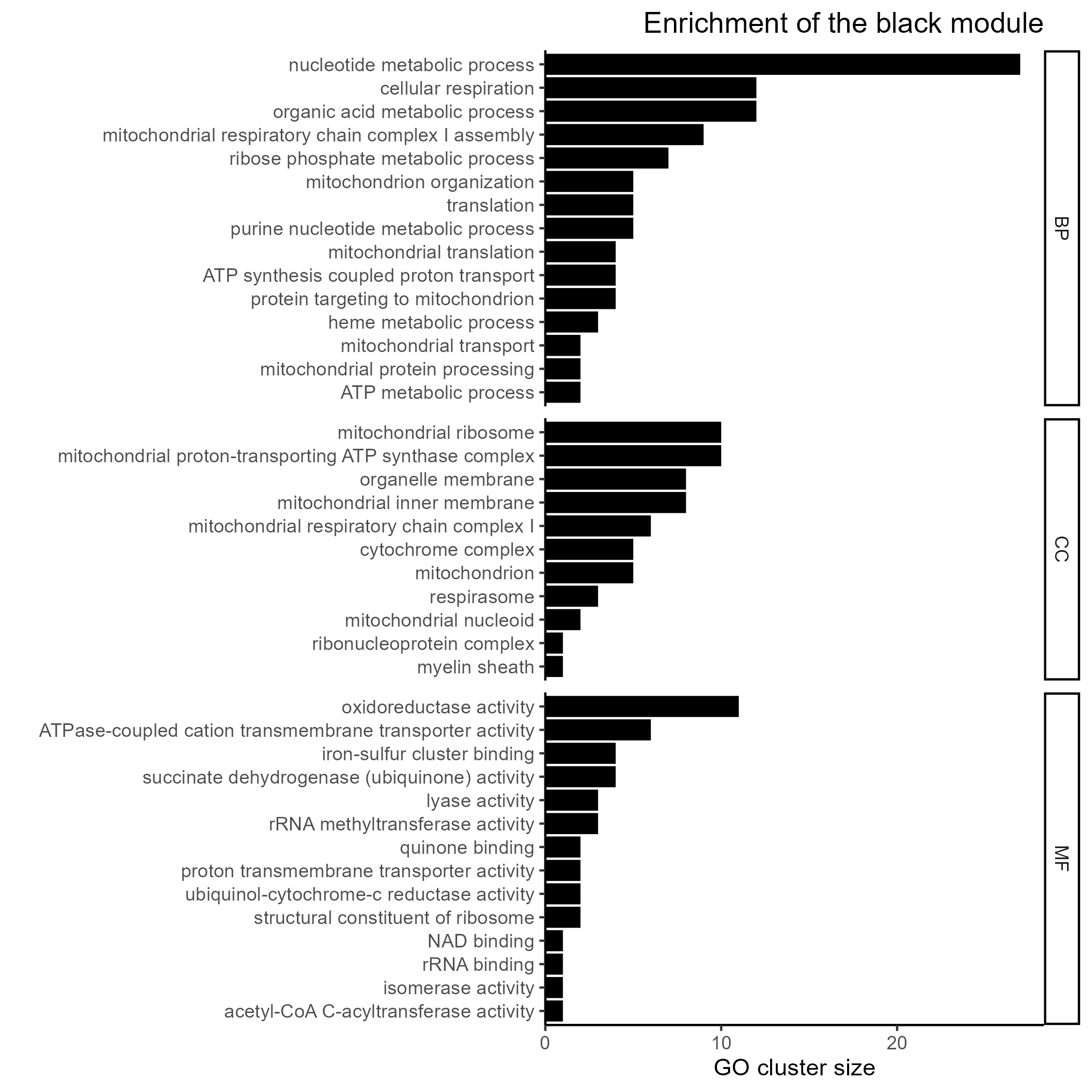

Supplement: Supplementary file 1 [file ijms-26-11572-s001.zip › 251120_U02_Supplementary/250819_SuppInfo_S3/enrich_black.png]

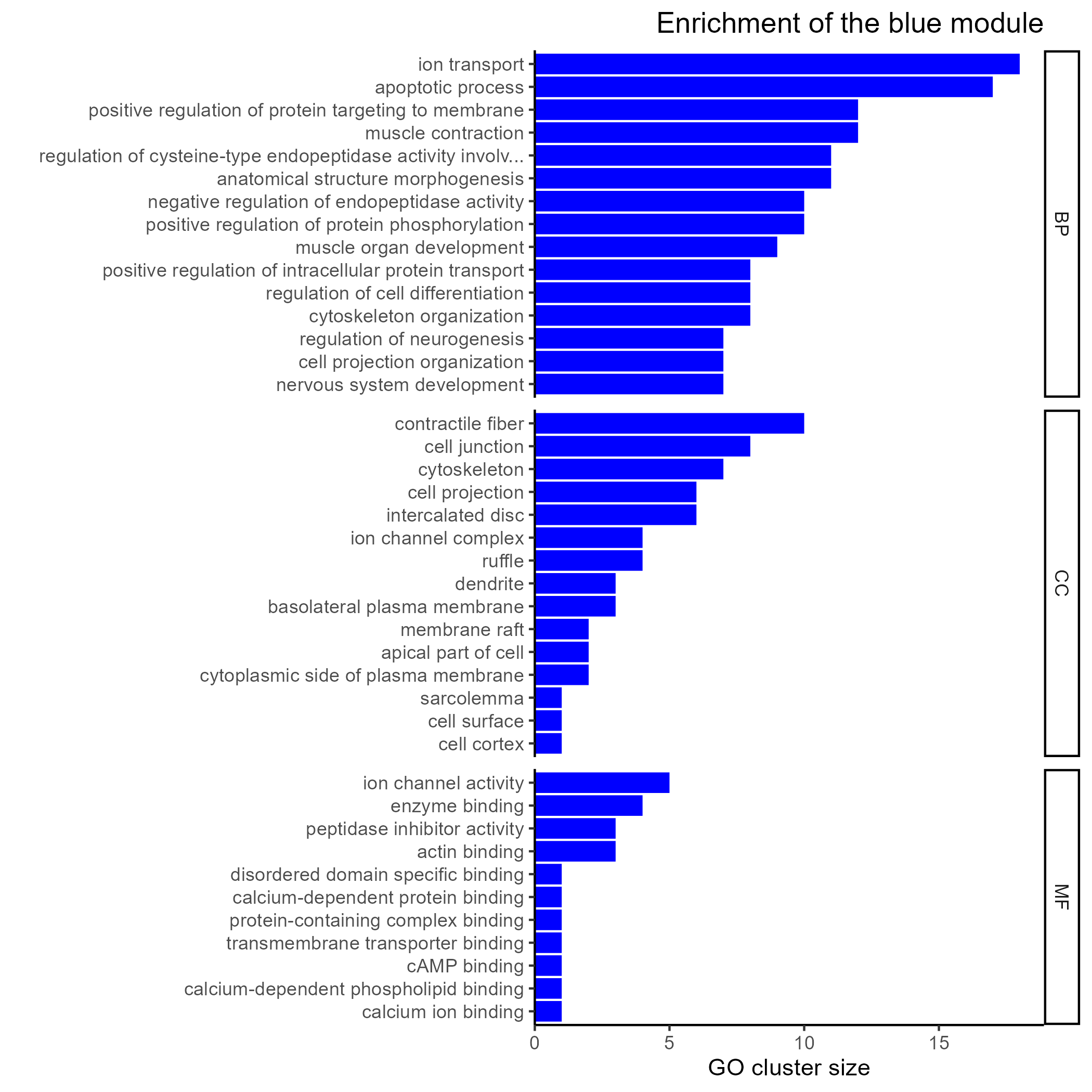

Supplement: Supplementary file 1 [file ijms-26-11572-s001.zip › 251120_U02_Supplementary/250819_SuppInfo_S3/enrich_blue.png]

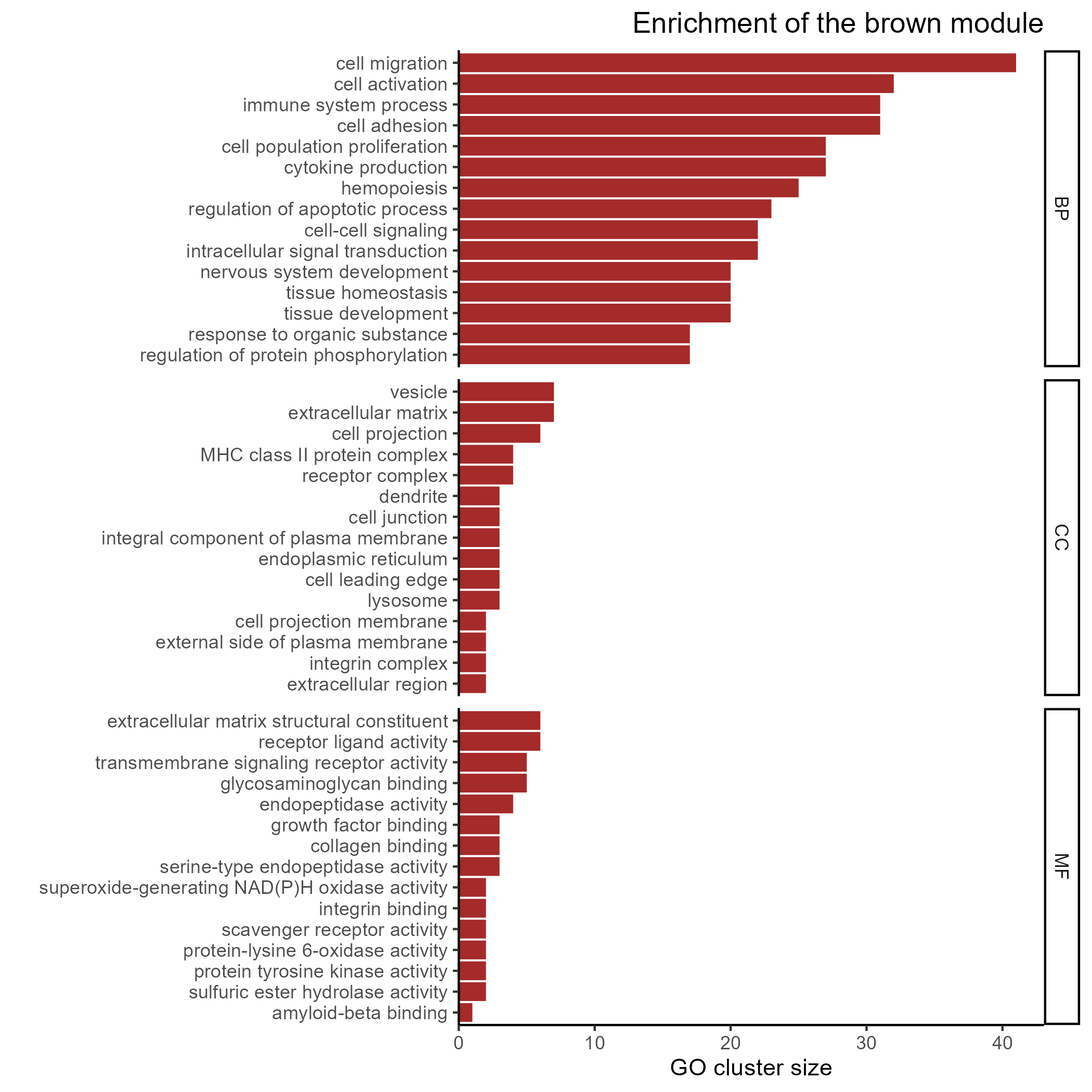

Supplement: Supplementary file 1 [file ijms-26-11572-s001.zip › 251120_U02_Supplementary/250819_SuppInfo_S3/enrich_brown.png]

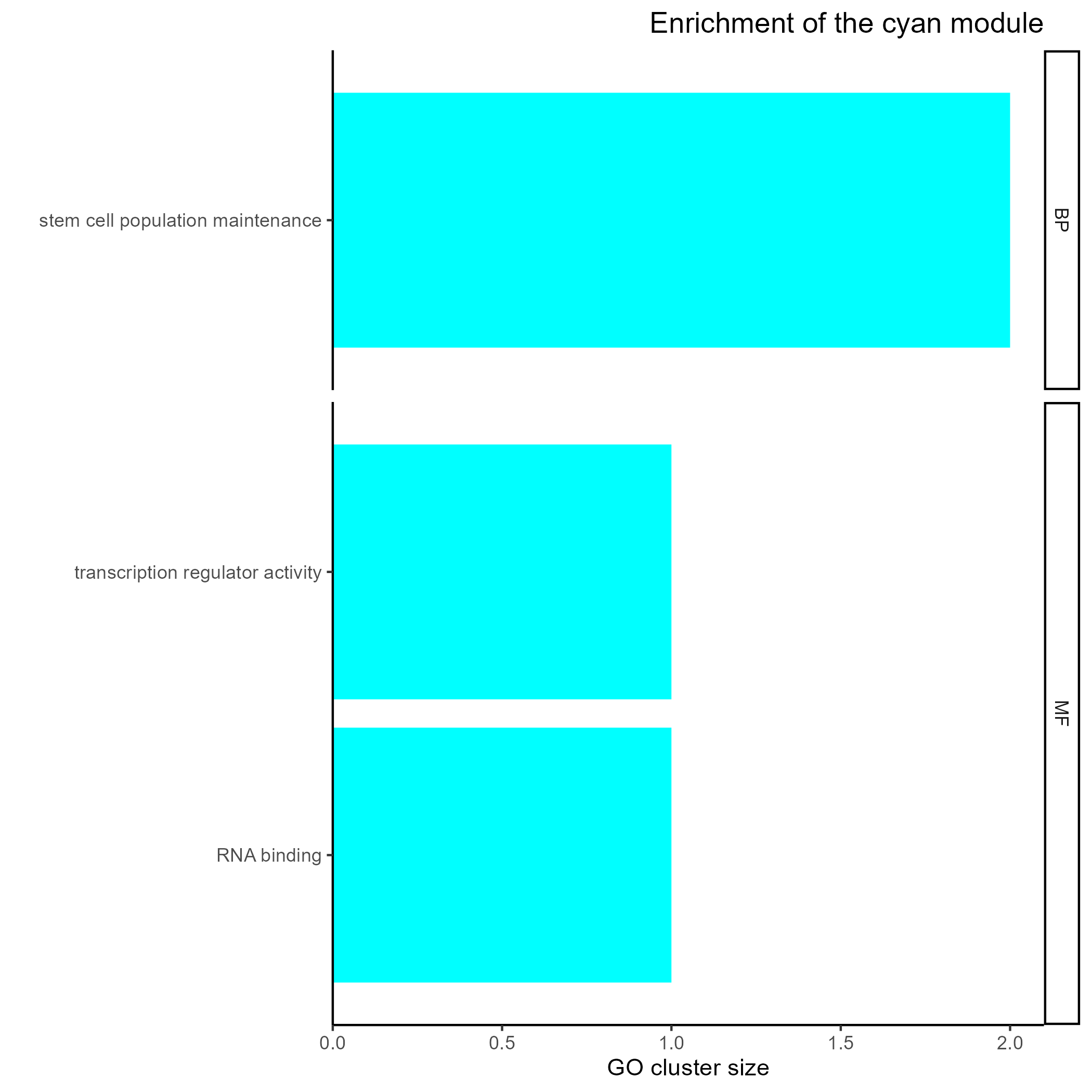

Supplement: Supplementary file 1 [file ijms-26-11572-s001.zip › 251120_U02_Supplementary/250819_SuppInfo_S3/enrich_cyan.png]

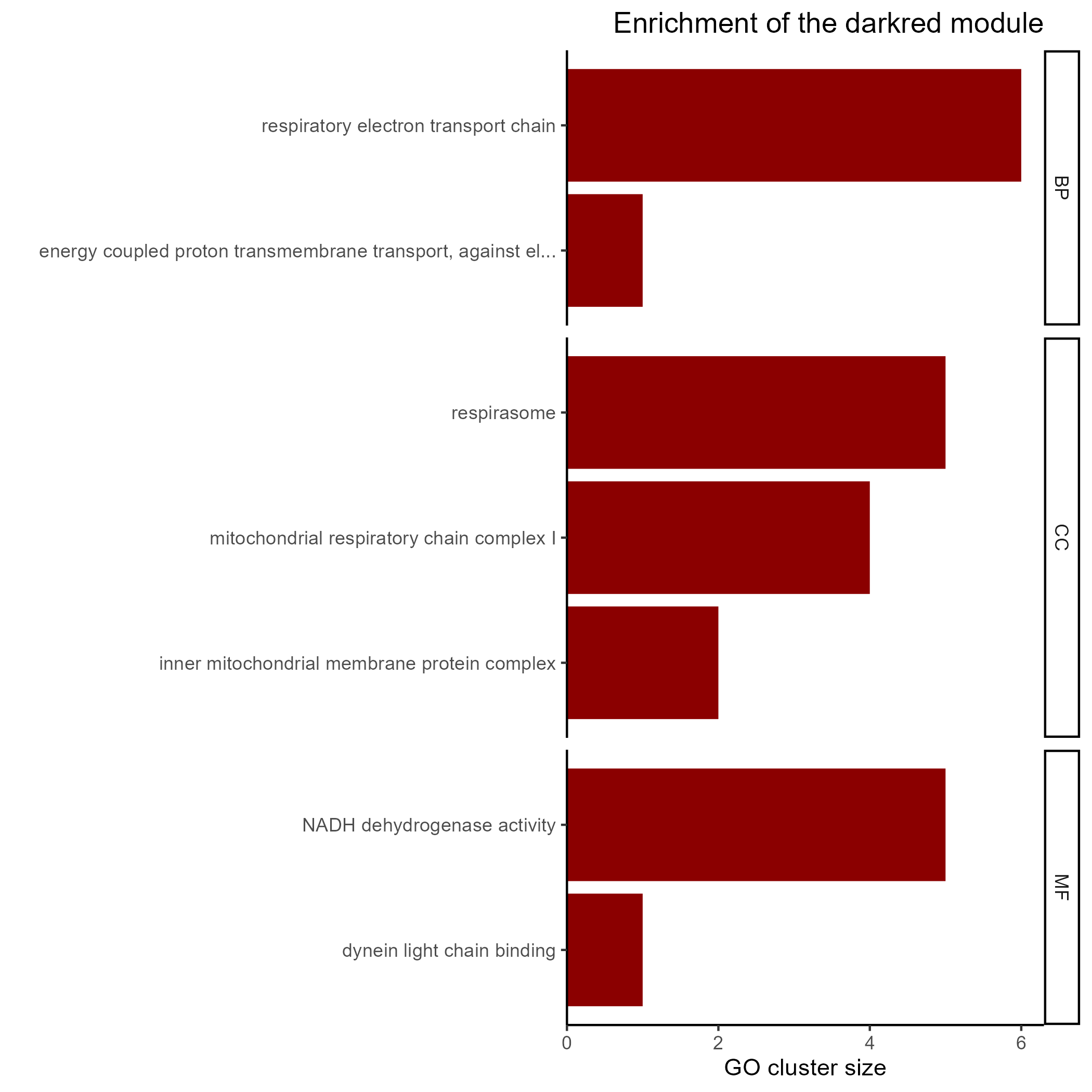

Supplement: Supplementary file 1 [file ijms-26-11572-s001.zip › 251120_U02_Supplementary/250819_SuppInfo_S3/enrich_darkred.png]

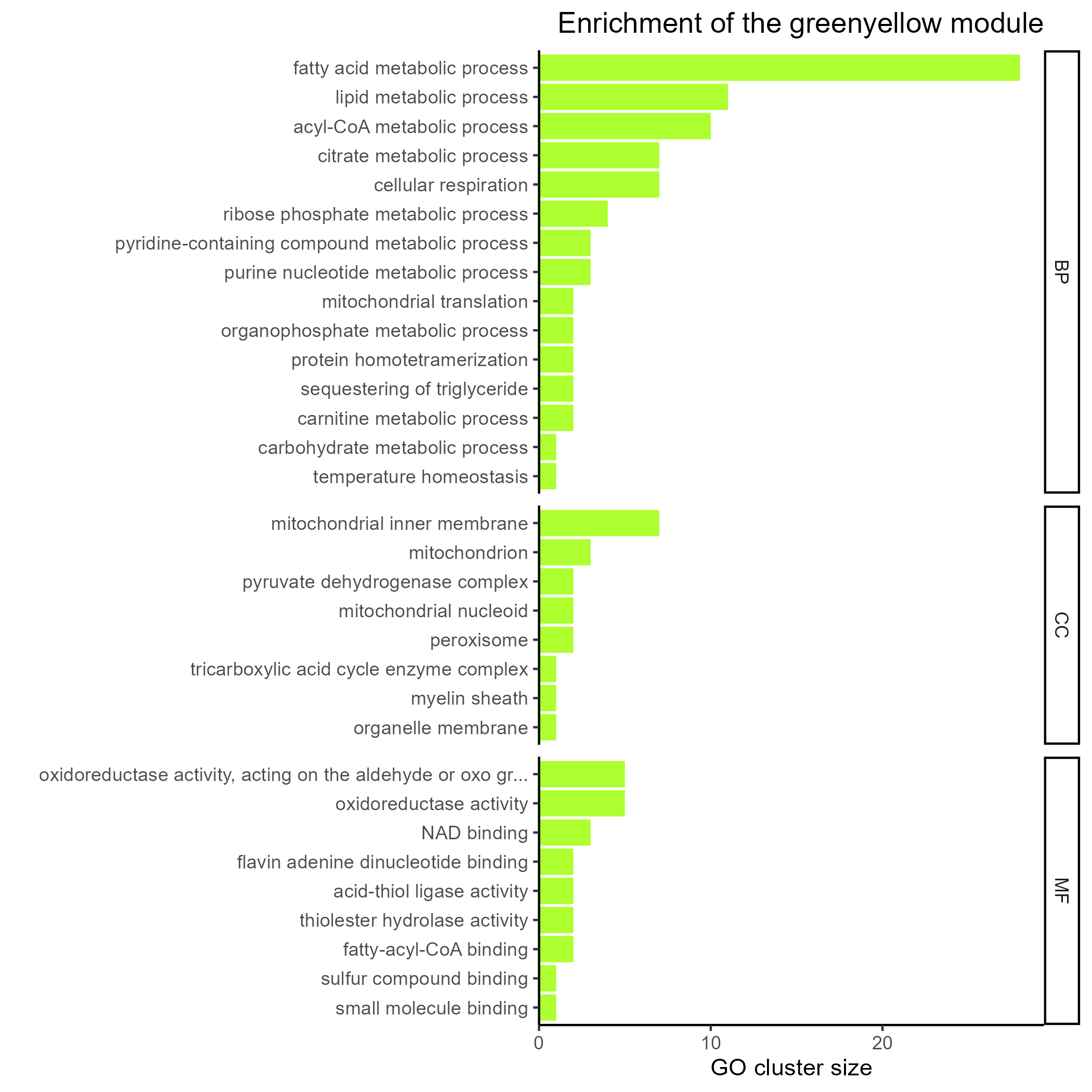

Supplement: Supplementary file 1 [file ijms-26-11572-s001.zip › 251120_U02_Supplementary/250819_SuppInfo_S3/enrich_greenyellow.png]

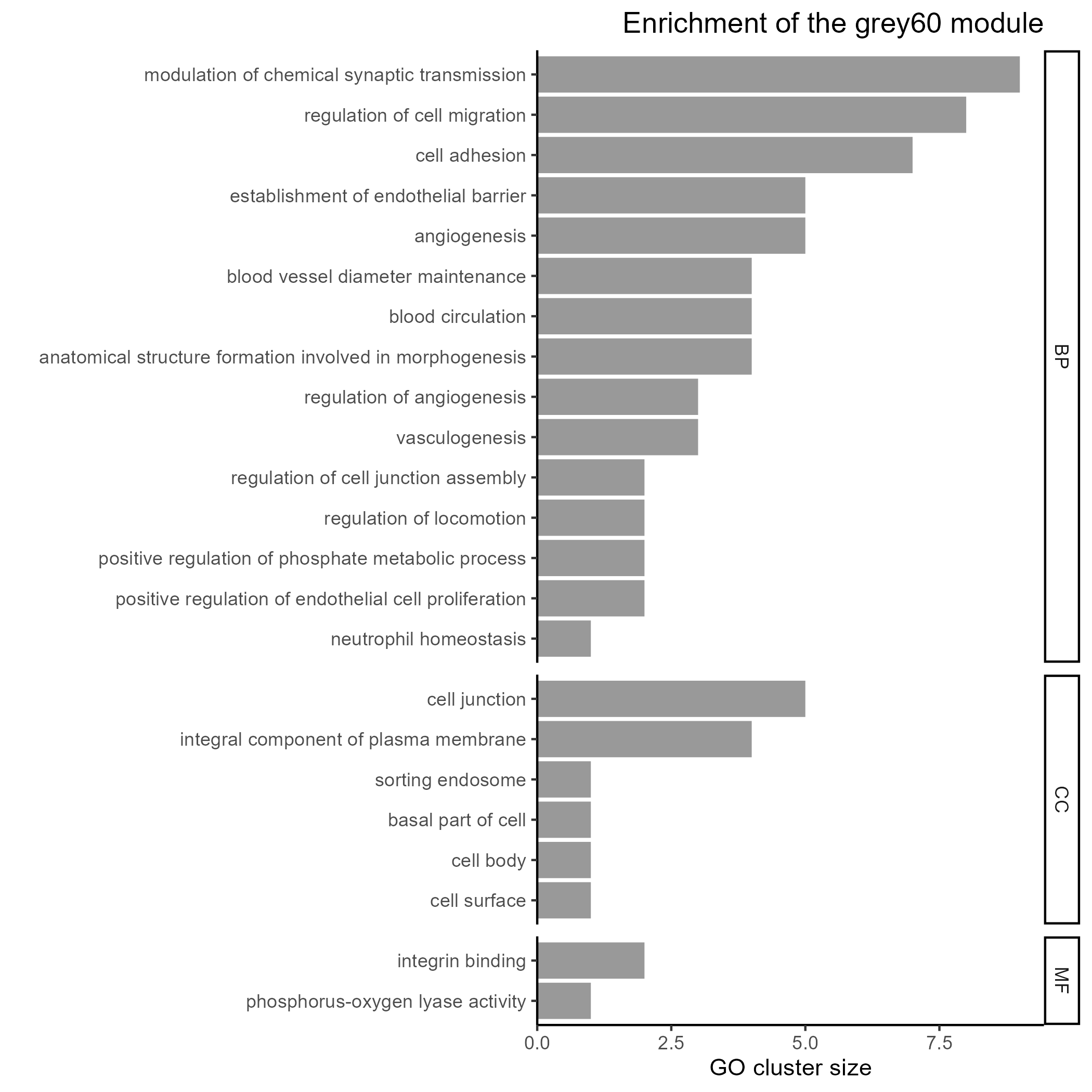

Supplement: Supplementary file 1 [file ijms-26-11572-s001.zip › 251120_U02_Supplementary/250819_SuppInfo_S3/enrich_grey60.png]

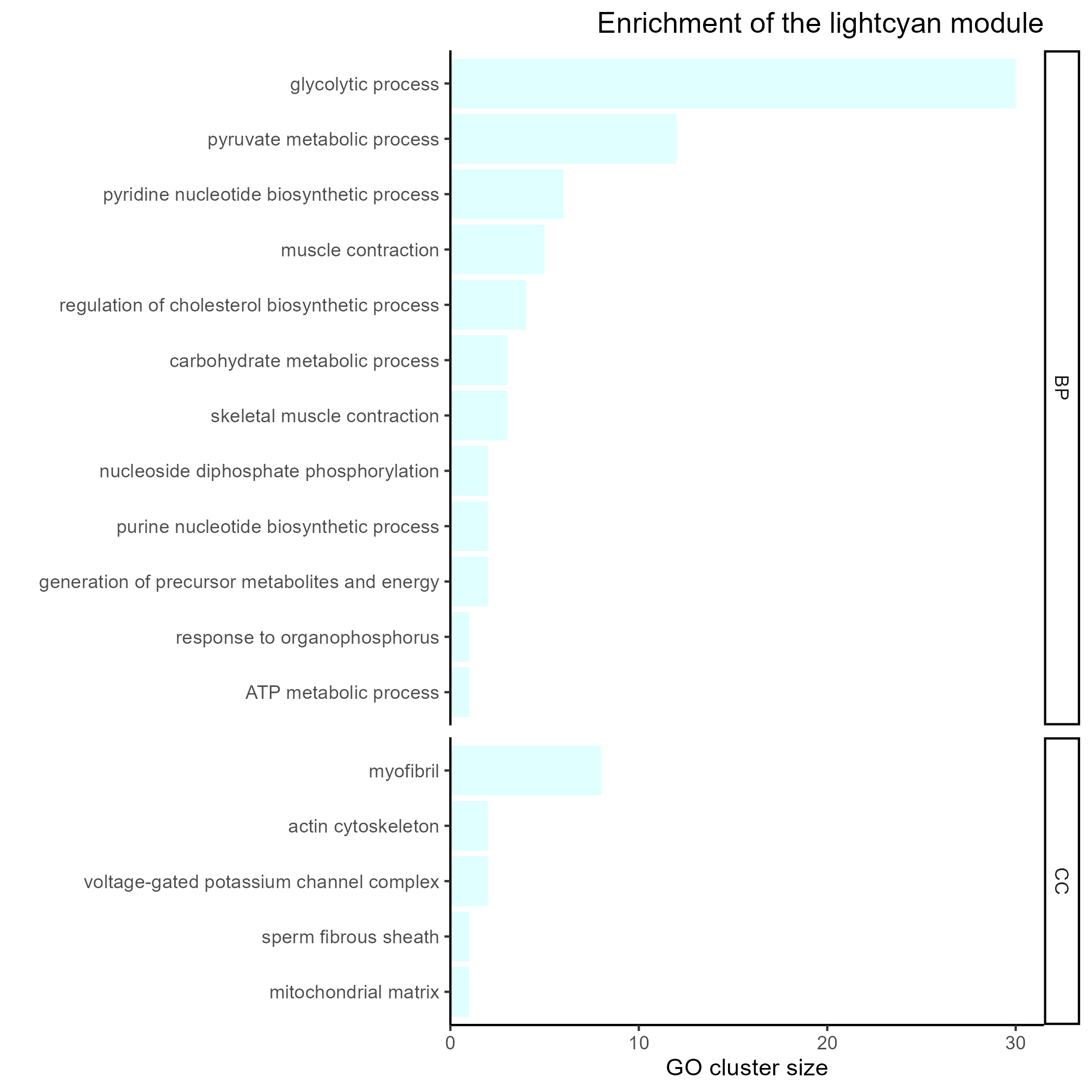

Supplement: Supplementary file 1 [file ijms-26-11572-s001.zip › 251120_U02_Supplementary/250819_SuppInfo_S3/enrich_lightcyan.png]

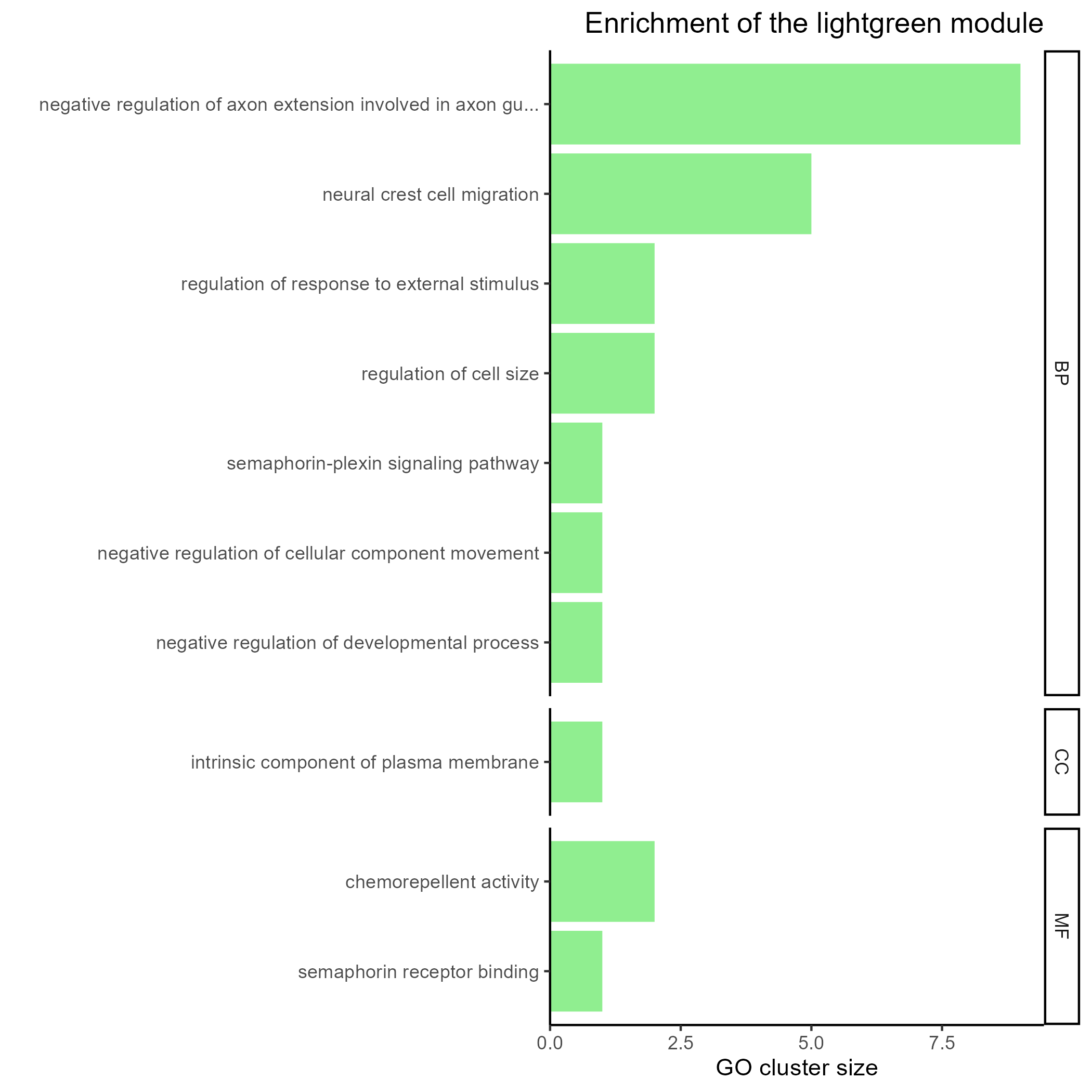

Supplement: Supplementary file 1 [file ijms-26-11572-s001.zip › 251120_U02_Supplementary/250819_SuppInfo_S3/enrich_lightgreen.png]

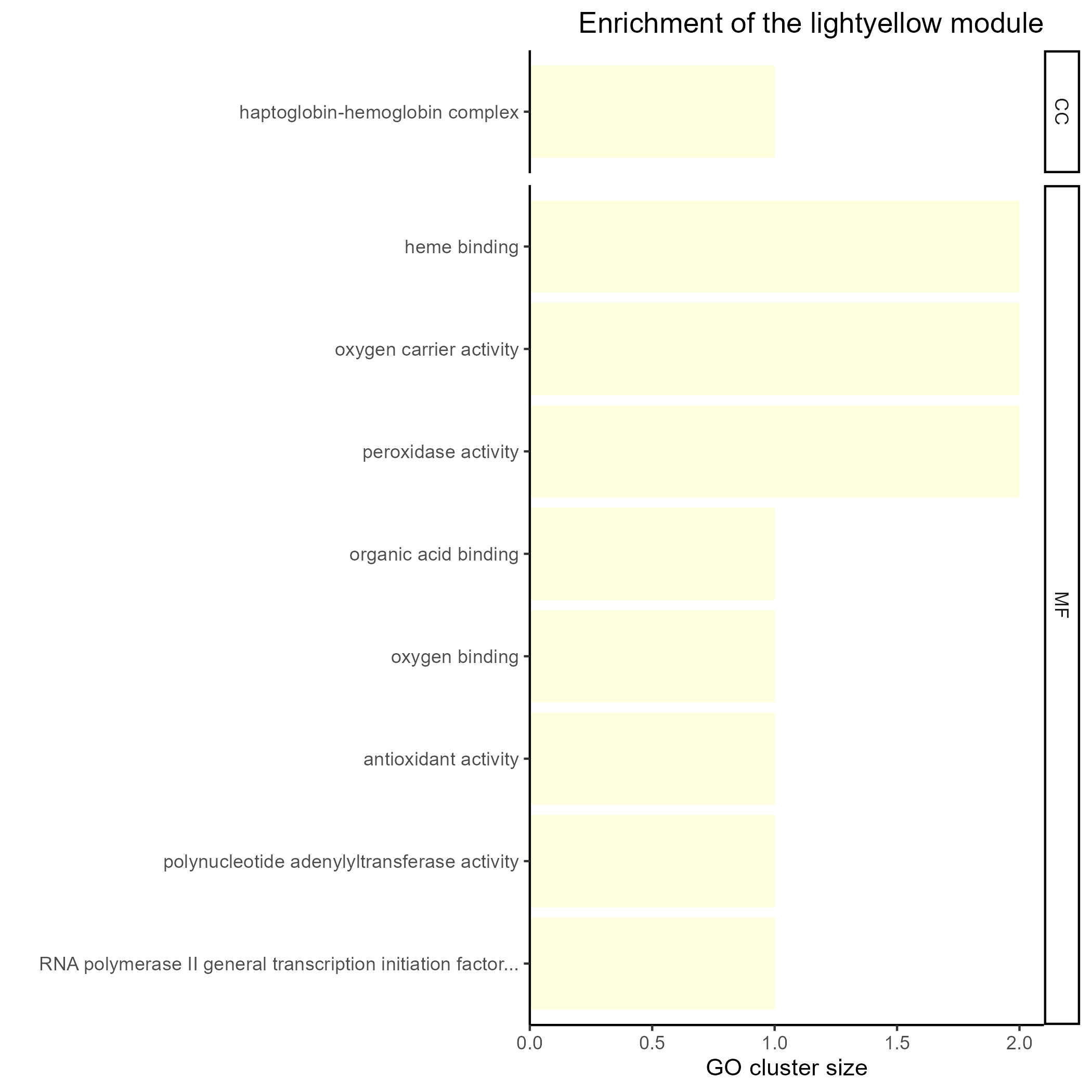

Supplement: Supplementary file 1 [file ijms-26-11572-s001.zip › 251120_U02_Supplementary/250819_SuppInfo_S3/enrich_lightyellow.png]

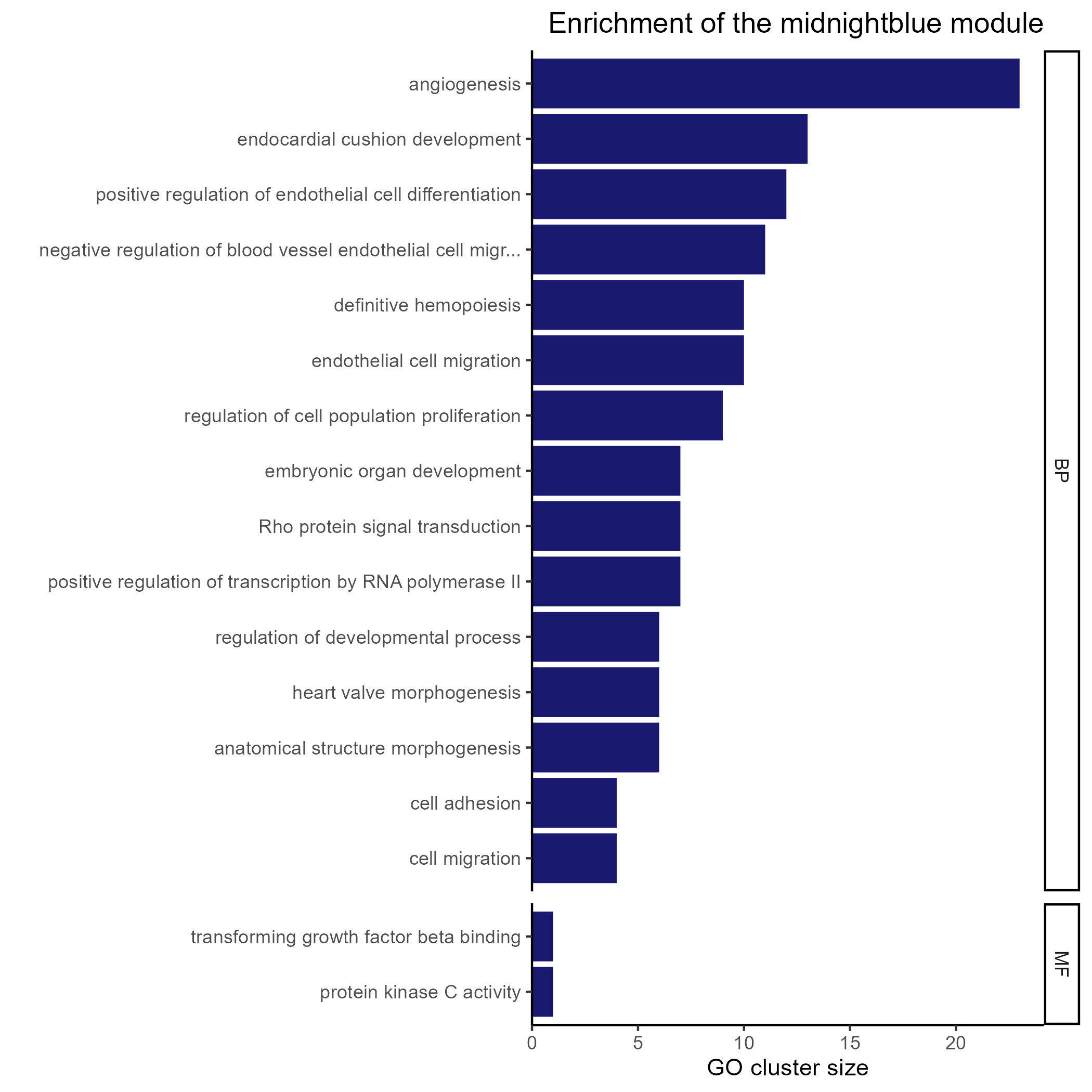

Supplement: Supplementary file 1 [file ijms-26-11572-s001.zip › 251120_U02_Supplementary/250819_SuppInfo_S3/enrich_midnightblue.png]

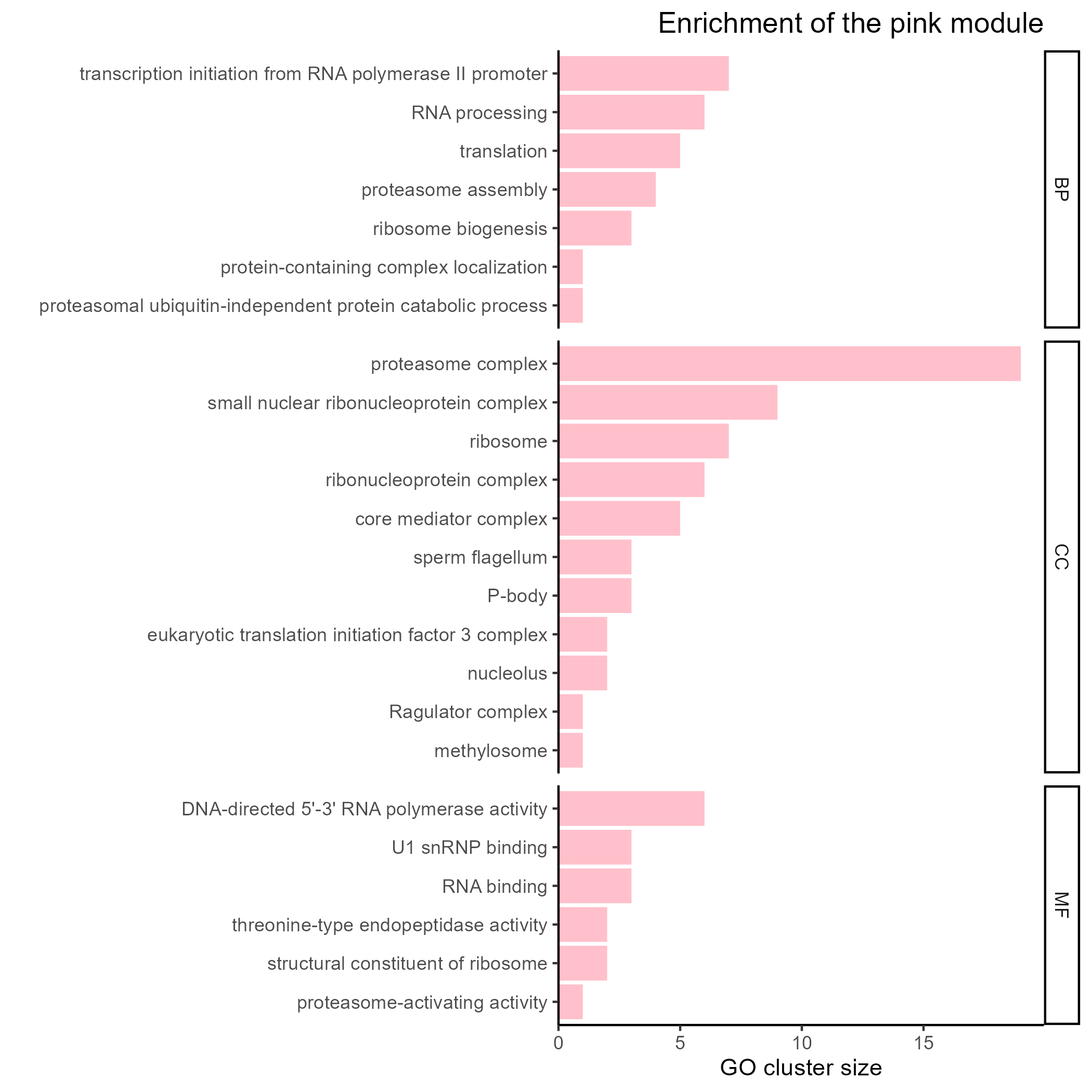

Supplement: Supplementary file 1 [file ijms-26-11572-s001.zip › 251120_U02_Supplementary/250819_SuppInfo_S3/enrich_pink.png]

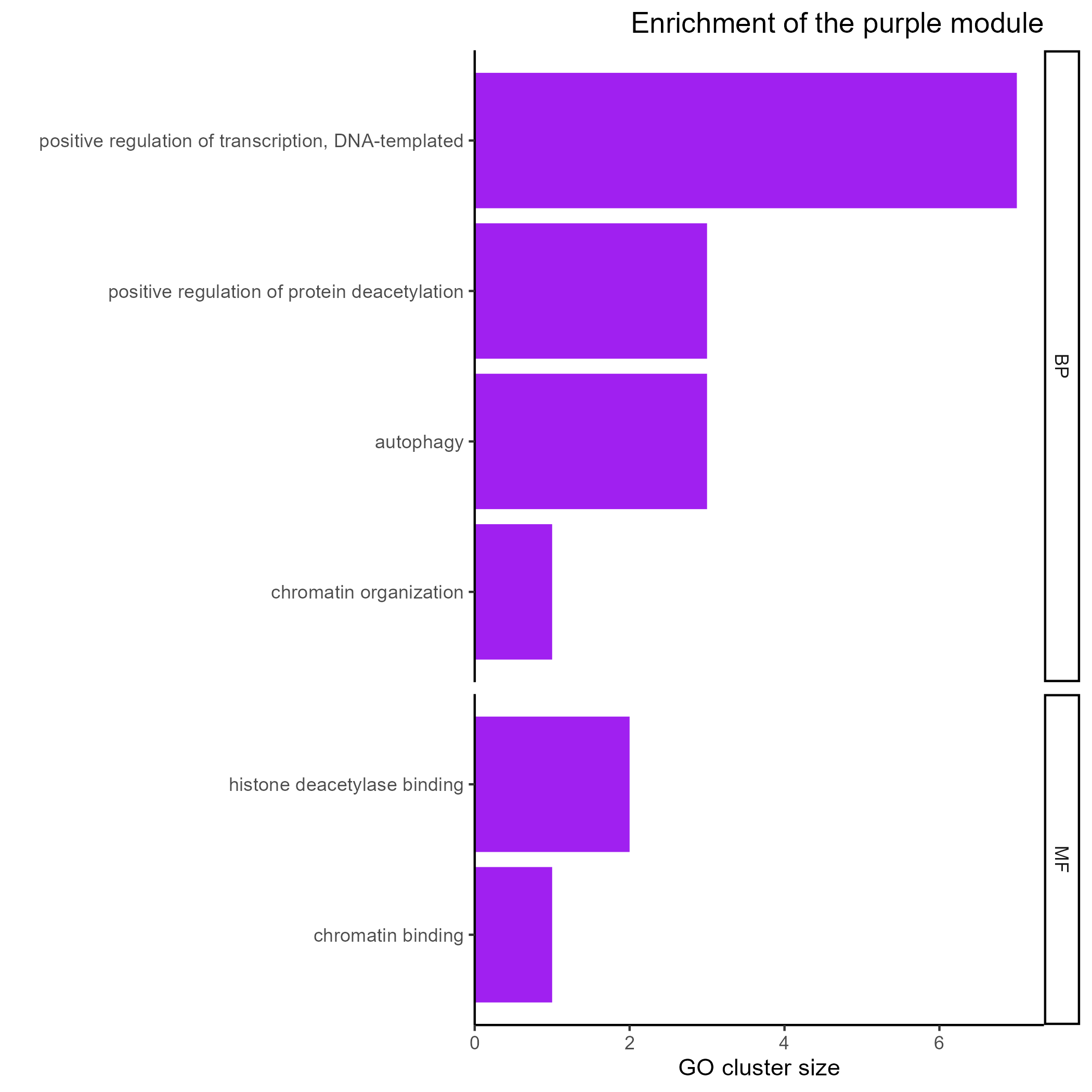

Supplement: Supplementary file 1 [file ijms-26-11572-s001.zip › 251120_U02_Supplementary/250819_SuppInfo_S3/enrich_purple.png]

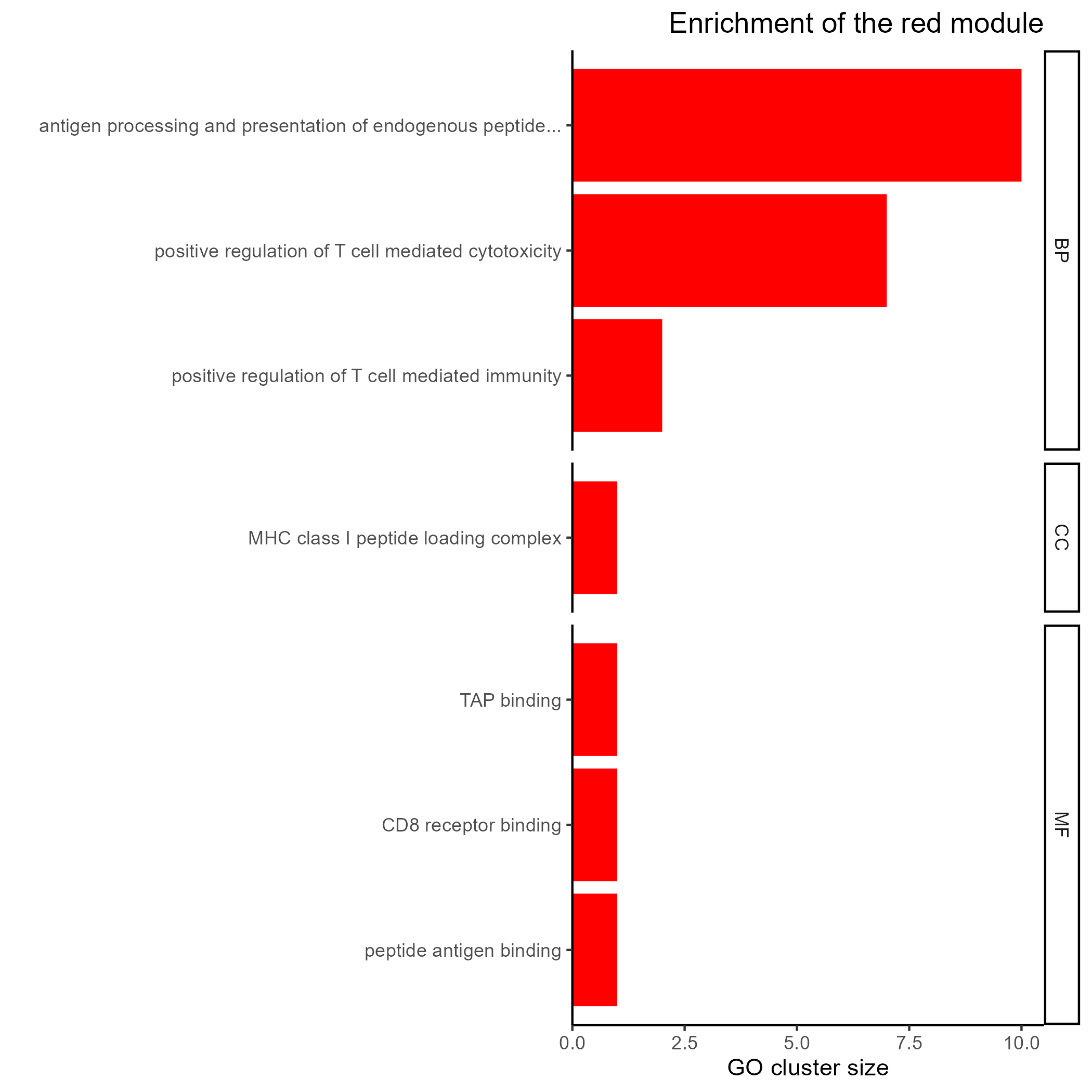

Supplement: Supplementary file 1 [file ijms-26-11572-s001.zip › 251120_U02_Supplementary/250819_SuppInfo_S3/enrich_red.png]

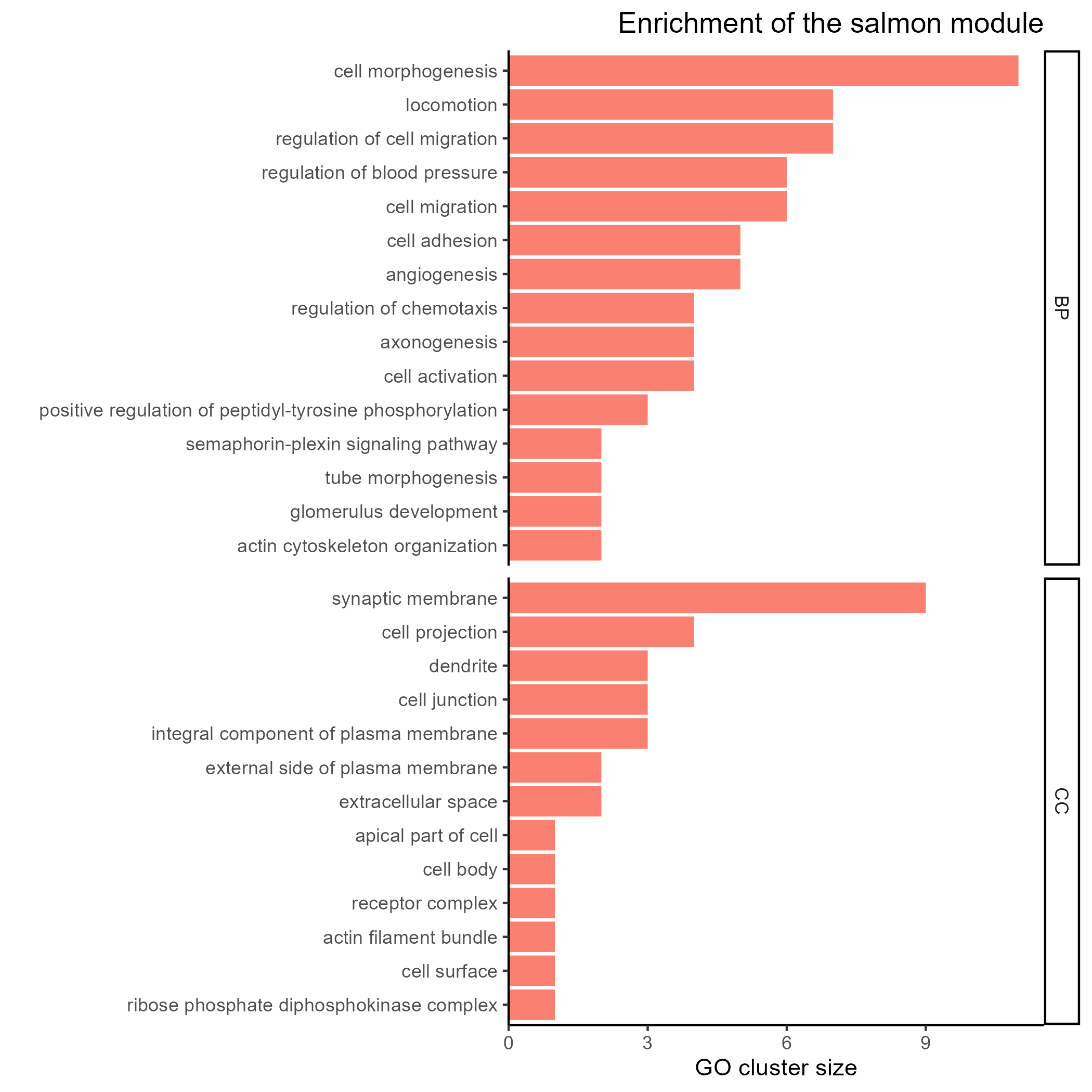

Supplement: Supplementary file 1 [file ijms-26-11572-s001.zip › 251120_U02_Supplementary/250819_SuppInfo_S3/enrich_salmon.png]

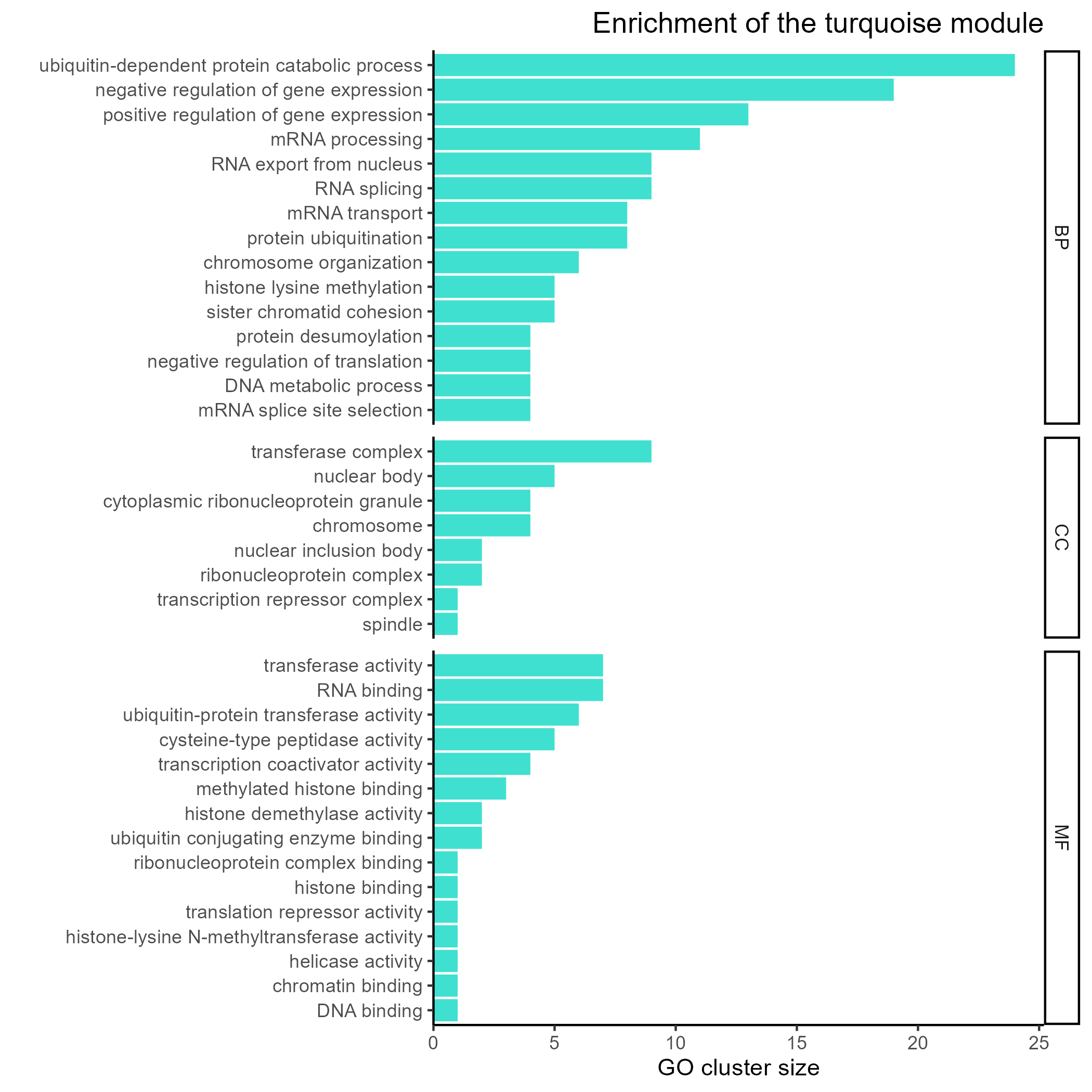

Supplement: Supplementary file 1 [file ijms-26-11572-s001.zip › 251120_U02_Supplementary/250819_SuppInfo_S3/enrich_turquoise.png]

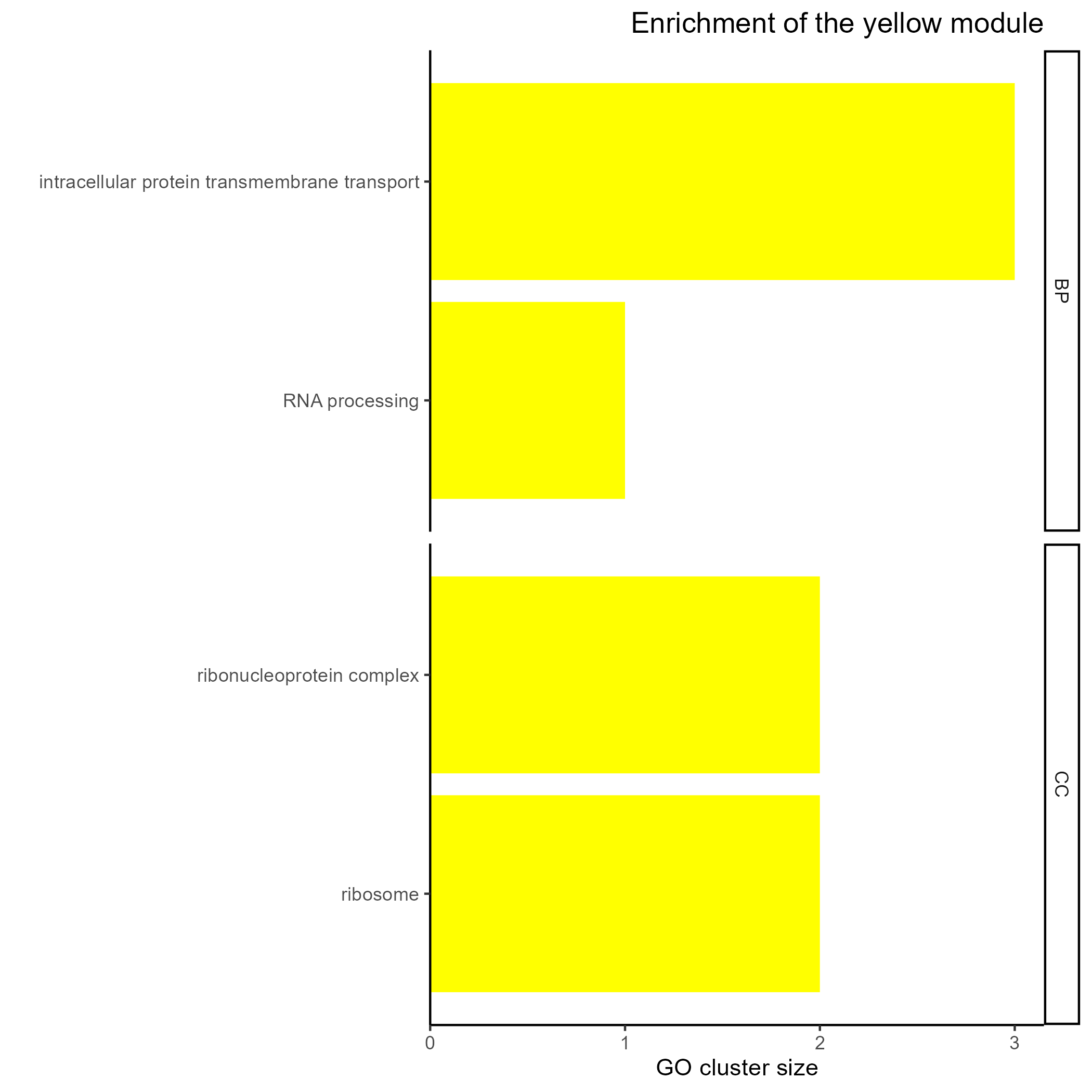

Supplement: Supplementary file 1 [file ijms-26-11572-s001.zip › 251120_U02_Supplementary/250819_SuppInfo_S3/enrich_yellow.png]

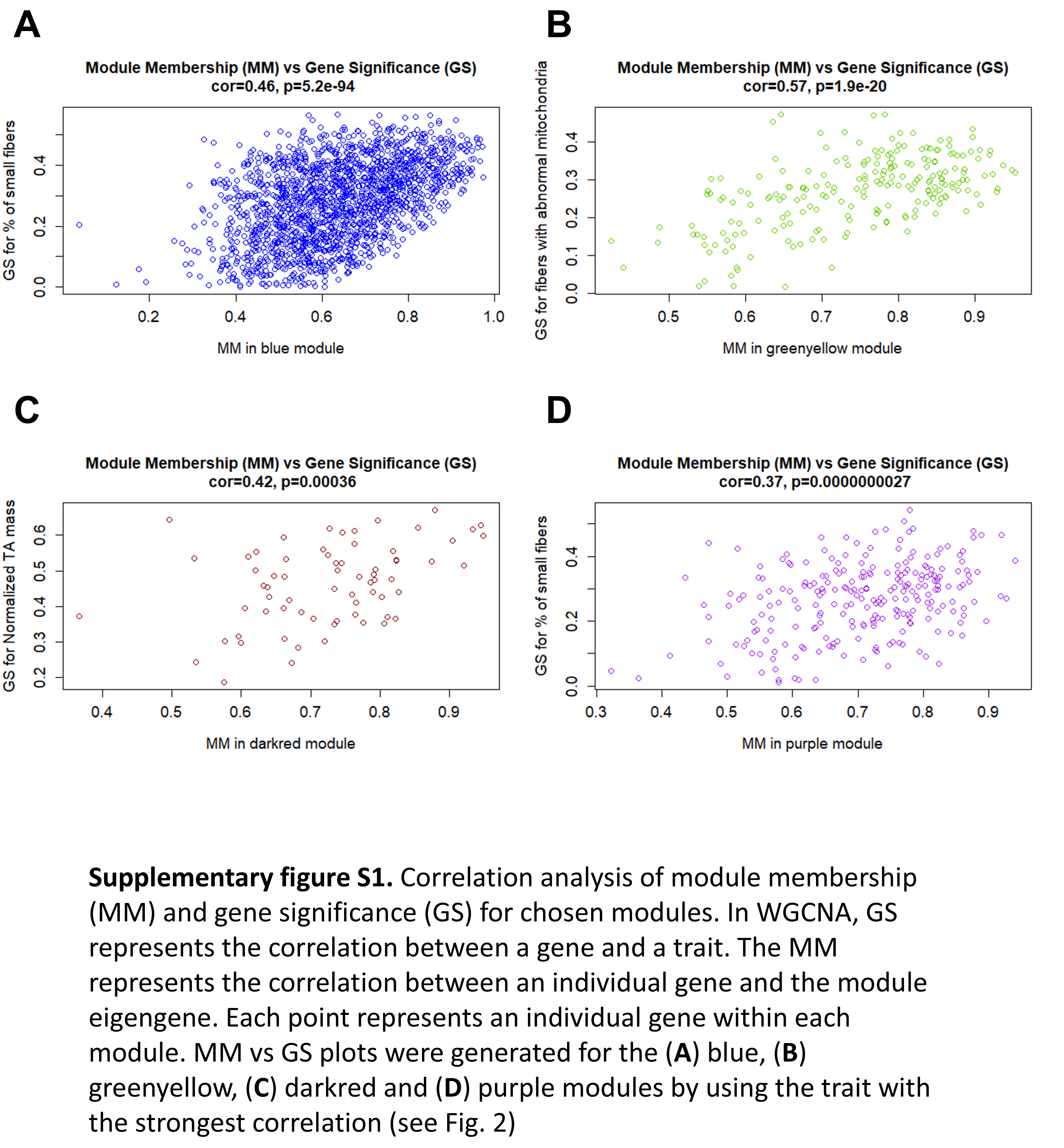

Supplement: Supplementary file 1 [file ijms-26-11572-s001.zip › 251120_U02_Supplementary/SuppFigure_S1.PNG]
